# A First Insight into North American Plant Pathogenic Fungi *Armillaria sinapina* Transcriptome

**DOI:** 10.3390/biology9070153

**Published:** 2020-07-04

**Authors:** Narimene Fradj, Nicolas de Montigny, Natacha Mérindol, Fatima Awwad, Yacine Boumghar, Hugo Germain, Isabel Desgagné-Penix

**Affiliations:** 1 Department of Chemistry, Biochemistry and Physics, Université du Québec à Trois-Rivières, 3351, boul. des Forges, Trois-Rivières, QC G9A 5H7, Canada; Narimene.Fradj@uqtr.ca (N.F.); Nicolas.De.Montigny@uqtr.ca (N.d.M.); Natacha.Merindol@uqtr.ca (N.M.); Fatima.Awwad2@uqtr.ca (F.A.); hugo.germain@uqtr.ca (H.G.); 2 Centre d’étude des Procédés Chimiques du Québec, 6220 rue Sherbrooke Est, Montréal, QC H1N 1C1, Canada; yboumghar@cmaisonneuve.qc.ca; 3 Groupe de Recherche en Biologie Végétale, Université du Québec à Trois-Rivières, 3351, boul. des Forges, Trois-Rivières, Québec, QC G9A 5H7, Canada

**Keywords:** fungi, transcriptomic, *Armillaria sinapina*, plant pathogen, RNA-Seq, white birch, betulin, de novo assembly, terpenoid, white rot disease

## Abstract

*Armillaria sinapina*, a fungal pathogen of primary timber species of North American forests, causes white root rot disease that ultimately kills the trees. A more detailed understanding of the molecular mechanisms underlying this illness will support future developments on disease resistance and management, as well as in the decomposition of cellulosic material for further use. In this study, RNA-Seq technology was used to compare the transcriptome profiles of *A. sinapina* fungal culture grown in yeast malt broth medium supplemented or not with betulin, a natural compound of the terpenoid group found in abundance in white birch bark. This was done to identify enzyme transcripts involved in the metabolism (redox reaction) of betulin into betulinic acid, a potent anticancer drug. De novo assembly and characterization of *A. sinapina* transcriptome was performed using Illumina technology. A total of 170,592,464 reads were generated, then 273,561 transcripts were characterized. Approximately, 53% of transcripts could be identified using public databases with several metabolic pathways represented. A total of 11 transcripts involved in terpenoid biosynthesis were identified. In addition, 25 gene transcripts that could play a significant role in lignin degradation were uncovered, as well as several redox enzymes of the cytochromes P450 family. To our knowledge, this research is the first transcriptomic study carried out on *A. sinapina*.

## 1. Introduction

In forest ecosystems, fungi represent a complex group of microorganisms that vary from uni- to multicellular entities. They are capable of developing in very different lifestyles, from saprophytes to plants, to animal and human pathogens, parasites and symbionts. Through their adaptative capacities, these organisms could play also roles as biocatalyst agents [1]. In addition, some fungi have the ability to interact with each other and with other ecosystem components in a multi-dimensional and complex way within the forest ecosystems [1,2,3,4].

Armillaria species are globally distributed in natural and planted forests of temperate, boreal and tropical ecosystems. Armillaria sp. are specific pathogens that colonize living trees and cause white rot. They are regarded among the most infamous and important phytopathogens of forest trees. More than 70 species have been listed worldwide, and more than 40 species of the genus Armillaria have been further studied [5,6,7]. In recent years, through the comparative genomic approach of over fifty fungi, the relationship between white rot fungi’s ability to excrete a number of extracellular enzymes involved in the degradation of wood polymers and other biotransformation processes was confirmed [8,9,10]. Armillaria species are saprotrophic and can, therefore, exploit all types of dead wood, such as roots and wood debris from different species in natural and artificial ecosystems. Thus, the mycelium of Armillaria spp. has the ability to break down cellulose and lignin, leading to the appearance of white rot [6].

*A. sinapina* was morphologically identified by J.A. Berubé and D. M. Dessureault in 1988 in North America and has been very poorly studied since then [11]. In a phylogenetic study based on the partial sequence of the translation elongation factor gene-1α (tef1) of several known and unknown Armillaria species from different regions of the world, *A. sinapina* from North America and from Japan clustered together as a distinct phylogenetic clade [12]. Armillaria species were previously described to infect forest-dominant species such as sugar maple (*Acer saccharum* Marsh.), northern red oak maple (*Quercus rubra* L.), beech (*Fagus grandifolia* Ehrh.), white ash (*Fraxinus americana* L.), black cherry (*Prunus serotina* Ehrh.), yellow birch (*Betula alleghaniensisis* Britton), and paper birch (*Betula papyrifera* Marsh.) [13]. Birch deaths were also associated with *A. sinapina* infection [14]. 

Terpenoids are interesting compounds, available in birch barks, presenting interesting properties that could be exploited. For example, triterpenoid antimicrobial betulin is highly concentrated in the birch’s outer bark of genus Betula (between 20–30%) [15], whereas anticancer betulinic acid is only present at low concentrations (1–2%) [16,17]. The sequential oxidation reactions involved in the conversion of betulin to betulinic acid has been identified and characterized in plants to be catalyzed by members of the cytochrome P450 monooxygenase (P450) family [18,19]. To date, no native fungal P450 involved in this conversion has been identified. However, a recent study reported that betulin has been successfully transformed into betulinic acid through biotransformation using *Armillaria luteo-virens* Sacc ZJUQH100-6 [20]. In the wild, growth and production of metabolites by *Armillaria spp.* is likely influenced by substrates provided by its host. North American *A. sinapina* is often found on birch known to have high concentrations of betulin [21], suggesting that it possesses enzymes able to degrade this host, but little is known. 

Knowledge on plant pathogenic fungi is benefiting from the increasing availability of genomic databases. In addition, recent advances in omics sciences have made possible the detection of peptides associated with plant–pathogen interactions and of the various post-translational changes observed during pathogenesis [7]. Transcriptome sequencing (RNA-seq) is a highly efficient, high throughput method used to characterize gene expression profiles and provides large amounts of genetic information. The development of high throughput platforms, such as Illumina RNA-seq, has allowed genomic expression profiles to be studied in various species and particularly non-model organisms. Due to its high sensitivity and specificity, as well as its ability to detect new genes, rare transcripts and new Single Nucleotides Polymorphisms, this technology can be used for associative investigations. Deep sequencing using RNA-seq, a NGS (next generation sequencing) technology, may be used to uncover gene function, differential gene regulation and metabolic pathways in the presence of several inducers in a given organism [22,23] or infer the genomic potential of species to carry out functions involved in infection strategies or substrate conversion.

Several genomic sequencing projects have been carried out on *Armillaria spp*. Up to now, six genomes of the Armillaria family have been sequenced (*A. cepistipes, A. gallica, A. fuscipes, A. mellea, A. ostoyae* and *A. solidipes*) [6,24,25,26,27,28]. However, the transcriptomic analysis of only one of the most virulent species, *A. solidipes* (also *A. ostoyae*), has been published [5,7].

The aim of this research was to characterize the transcriptome of the fungal mycelium of the boreal forest species *A. sinapina*. Next, we investigated the influence of betulin on *A. sinapina* transcriptome to better understand the genetic response to this compound, as well as to determine its impact on the enzymes involved in degradation of the cell walls of birch bark, and to potentially discover new genes involved in the biotransformation of betulin into betulinic acid, in an effort to valorize forest residues.

## 2. Materials and Methods

### 2.1. Fungal Culture and Growth Conditions

*A. sinapina* fungal culture was provided by the Biopterre laboratory (La Pocatière, QC, Canada). *A. sinapina* mycelium was maintained on Potato Dextrose Agar (PDA) medium, and stock cultures were stored at 4 °C. The identity of the *A. sinapina* isolate was confirmed by sequencing of the ribosomal internal transcribed spacer (ITS) DNA region, which displayed 100% sequence identity to *A. sinapina* isolate XY17_48 ITS NCBI sequence ID MH550358.1. To optimize the mycelial growth of *A. sinapina*, the culture medium of Yeast Malt Agar (YMA) was selected [20,29,30,31,32]. Different growth condition parameters of liquid and solid culture of *A. sinapina* were tested in the presence or absence of betulin (15 µg/mL), as described previously [32].

### 2.2. Isolation of RNA 

RNA was isolated from three biological replicates of the cultured mycelium of *A. sinapina* according to Fradj et al. [32]. Briefly, RNA was extracted from *A. sinapina* liquid culture of YMB medium supplemented with 0.2% DMSO (control) or 15 µg/mL of betulin (BET) and grown for 8 days on a rotary incubator at 150 rpm and 28 °C in the dark. The mycelium was filtered, frozen with liquid nitrogen, and then, placed in a 2 mL tube with 2 mL of TRIzol reagent (Fisher Scientific, Ottawa, ON Canada). The mycelia were crushed using 5 mm diameter stainless steel beads with the TissueLyser II (QIAGEN, Qiagen Retsch GmbH, Hannover, Germany) at a speed of 30 strokes per second for 3 min until a homogeneous sample was obtained. Tubes were incubated on ice for 5 min, then 400 µL of chloroform was added. Tubes were vigorously vortexed for 15 s and incubated in an ice bath for 3 min. After centrifugation at 13,000× *g* for 15 min at 4 °C, two phases were obtained; the clear upper phase of the supernatant, exempt of cellular debris, was recovered and transferred into a clean 1.5 mL microtube. RNA was concentrated using isopropanol [32]. RNA pellets were resuspended in 20 μL of nuclease-free water. RNA quality and quantity were determined based on absorbance ratios using the Nanodrop and by the Bioanalyzer (Agilent Technologies, Inc., Santa Clara, CA, USA). Samples with an RNA integrity number higher than 8 were selected for Illumina sequencing.

### 2.3. Transcriptome Sequencing, De Novo Assembly and Functional Annotation

Sequencing was performed using Illumina HiSeq 4000, PE 100 paired ends, at McGill University (Montreal, QC, Canada) and Genome Québec Innovation Centre (Montreal, QC, Canada) on cDNA libraries converted from isolated high quality mRNA. Low quality reads, reads with unassigned nucleotides and adapters were filtered out. Trimmomatic was used to remove the adapters, trim reads from the 3’ end and filter all reads below 50 bp in order to obtain clean reads [33]. Then, normalization was performed to remove redundant reads in datasets without affecting its Kmer content [34]. De novo assembly of cleaned and standardized reads was performed using the Trinity (v2.6.5) assembler [35]. This method assembles the RNA-seq reads into full-length transcripts, called contigs or unigenes. The predicted unigenes were aligned and searched in the uniprot_sprot.trinotate_v2.0.pep public protein database using the BLASTX algorithm with an E-value cutoff threshold of 10-5 against the NCBI non-redundant (nr) protein database. Top BLAST hits were used for annotation of component/gene for each transcript. To quantify the gene transcript abundance, the raw RNA-Seq reads were mapped to assembled transcripts with Bowtie using the default parameters [36]. The gene transcripts’ abundance was calculated as transcripts per kilobase million (TPM) [37,38].

Functional annotation of unigenes was performed using Trinotate (http://trinotate.github.io/), by aligning transcripts to the Swiss Institute of Bioinformatics databases (Swiss-Prot) with BLASTX and identifying protein domains (HMMER/PFAM), protein signal peptides, transmembrane domains prediction (signalP/tmHMM), and Clusters of Orthologous Groups (COG). Then, the functional annotation of unigenes was compared to curated annotation databases (EMBL UniProt eggNOG/GO Pathways databases). The Trinity assembly and the functional annotation of unigenes were integrated as an annotation report into an SQLite database.

Gene Ontology (GO) enrichment analysis for the unigenes was performed using Swiss-Prot. The WEGO 2.0 software was used to obtain GO functional classification and OmicsBox v.1.2.4 for the Kyoto Encyclopedia of Genes and Genomes (KEGG) pathway analyses for all unigenes. The raw reads under the accession number PRJNA565538 were deposited to the Sequence Read Archive [39].

### 2.4. Accession Numbers

The sequences described in this paper have been deposited in the National Center for Biotechnology Information Sequence Read Archive (https://www.ncbi.nlm.nih.gov/sra/) under the accession number PRJNA565538.

### 2.5. Statistical Analysis

Modulations of transcripts levels with adjusted P-values ≤ 0.05 and a fold change (log2FC) ≥ 1 were designated as statistically significant differentially expressed genes (DEG). Differential analysis was done by Genome Québec with “DESeq2”, using raw counts with “R” and its packages as for *Inonotus obliquus* and KEGG, which are identified with OmicsBox, which uses UniProt and KEGG databases [32].

## 3. Results

### 3.1. Illumina Sequencing and De Novo Assembly

**Figure 1:**
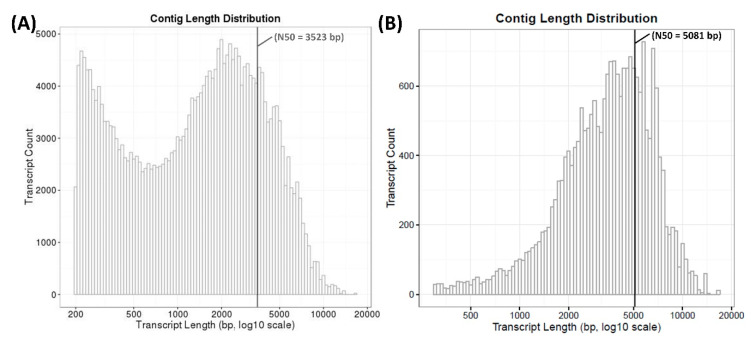
Sequence length distribution of transcripts of *A. sinapina* after Trinity de novo assembly (**A**; N50 = 3523bp) and after BLAST annotation and filtered annotated components (**B**; N50 = 5081bp).

To better understand the molecular mechanisms underlying the differences in *A. sinapina*’s transcriptome caused by the presence of betulin, RNA-Seq and de novo assembly were performed. RNA was extracted from biological replicates of cultured *A. sinapina* in the absence (control) or presence of the terpenoid substrate betulin (BET). A total of 170,592,464 raw paired reads were generated from all replicates (both conditions were analyzed together, Table 1). After filtering out low quality sequences, 166,006,772 clean reads were obtained, corresponding to approximately 97.3% of the total raw reads (Table 1). In view of the lack of availability of information (i.e., genome, transcriptome data) on *A. sinapina*, all clean reads obtained from the libraries were de novo assembled using Trinity. A total of 273,561 transcripts with an average length of 2051 bp, and a N50 length of 3523 bp were obtained (Table 1; Figure 1A). An evaluation of the size distribution showed that 96% of all annotated transcripts of *A. sinapina* measured more than 1 kb (Figure 1B).

### 3.2. Functional Annotation of A. Sinapina Transcriptome

A total of 121,959 transcripts (44.6%) homologous to known proteins or conserved hypothetical proteins were obtained from the search using BLASTX. Next, 103,267 (37.75%), 61,272 (23.53%), 45,683 (16.70%), and 105,195 (40.39%) transcripts matched annotated sequences from the GO, COG, Pfam and KEGG databases, respectively. A total of 145,191 (53.07%) transcripts were annotated in this way (Table 2). Strikingly, 128,370 (46.92%) transcripts showed no similarity with known sequences from the databases, which suggests that unknown new genes have been uncovered from *A. sinapina*.

**Figure 2:**
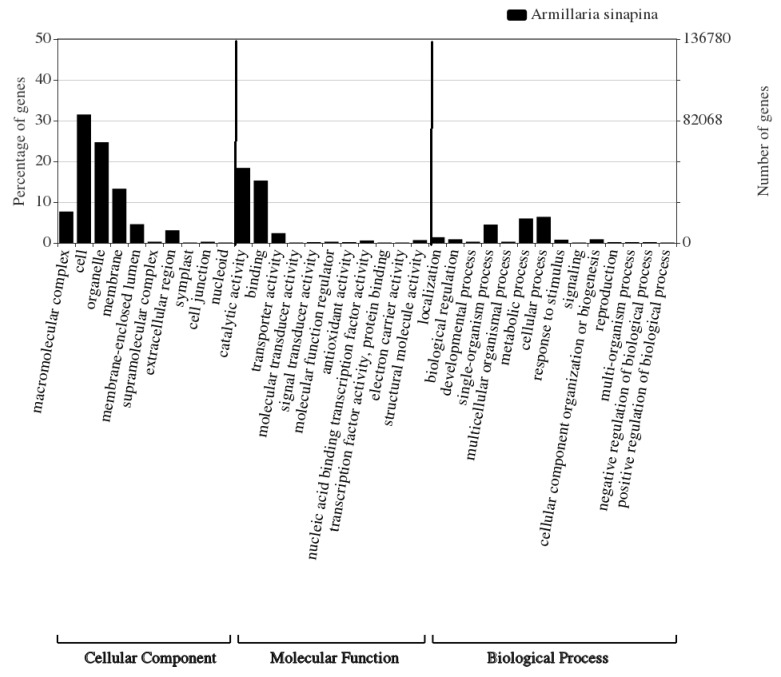
Gene Ontology (GO) terms of 35 functional groups of expressed transcripts from *Armillaria sinapina.*

Of the 103,267 transcripts matched to known GO terms (Figure 2), the most abundant GO category was ‘cellular component’ with 48.0% (95,002 transcripts), followed by ‘molecular function’ with 39.1% (77,267 transcripts), and lastly, ‘biological process’ with 12.9% (25,492 transcripts). GO slim terms for molecular function included catalytic, binding, and transporter activities, reflecting the ability of *A. sinapina* to transport and metabolize diverse compounds. Cellular component terms consisted of 10 groups, in which the top three were cell, organelle and membranes (Figure 2).

**Figure 3:**
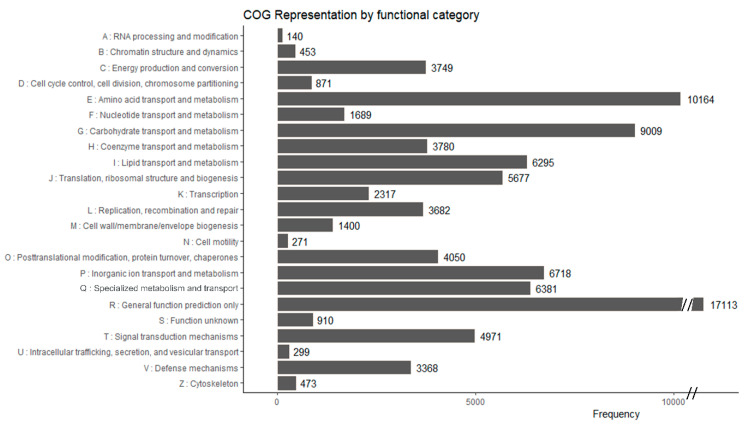
Functional classification of *Armillaria sinapina* transcriptome using the Cluster of orthologous groups (CoG).

The 61,272 (23.52%) unigenes collected from COG were classified into 25 categories (Figure 3). A large number of transcripts (17,113) were classified in the functional category R (general function prediction only), followed by category E (amino acid transport and metabolism; 10,164 transcripts), and category G (carbohydrates transport and metabolism; 9009 transcripts) (Figure 3). Interestingly, the functional category specialized metabolism, transport and catabolism (Q), represented 8% (6381 transcripts) of the COG annotated transcripts.

**Figure 4:**
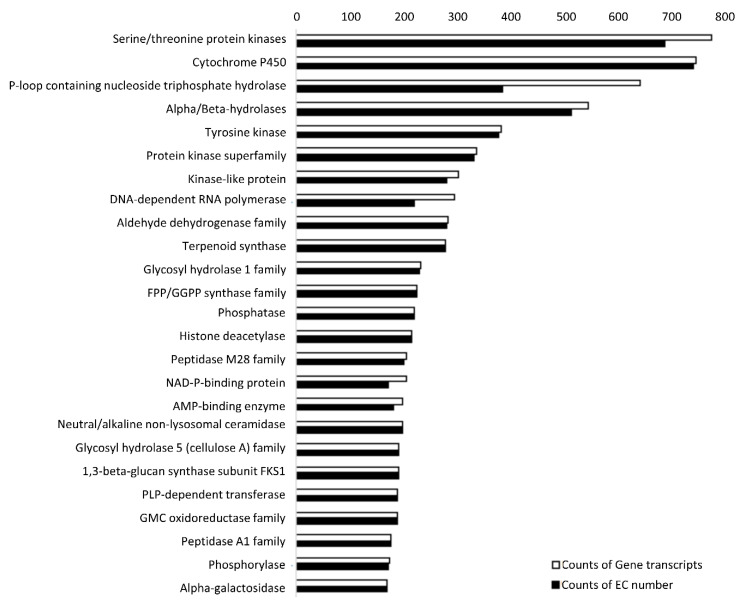
Annotation of the *Armillaria sinapina* putative proteins using the Kyoto encyclopedia of genes and genomes (KEGG).

Furthermore, a total of 105,195 (40.39%) transcripts from *A. sinapina* transcriptome were assigned using the pathways reported in the KEGG (Figure 4). Metabolism, genetic information processing, environmental information processing, cellular processes, and organismal systems were present. The most highly represented category of KEGG function was “the global and overview maps”. Genes encoding specialized metabolite biosynthesis, carbohydrate metabolism, energy metabolism, lipid metabolism and nucleotide metabolism were also uncovered. These results indicate that active metabolic processes were ongoing in *A. sinapina*. Altogether, the results for GO, COG, Pfam and KEGG annotations suggest that most of the transcripts expressed in *A. sinapina* cultured cells took part in basic biological processes, such as metabolism and biological regulation.

### 3.3. Differential Expression Analysis

Differential expression analyses were performed between the generated databases of *A. sinapina*. The addition of betulin during *A. sinapina* cells culture led to 3943 differentially expressed genes (DEG). Forty-eight times more transcripts (3863) were up-regulated than down-regulated (80) in the presence of betulin (control vs. BET) (Table 3). This suggests that *A. sinapina* undergoes a transcriptomic adjustment in the presence of botulin and activates the expression of a significant number of genes. In the top 50 up-regulated transcripts, several transcripts encoded proteins involved in lipid and carbohydrate metabolism such as ketoacyl-CoA thiolase, phosphoglycerate kinase, pyruvate decarboxylase, fumarate reductase, glycoside hydrolase, phosphopyruvate hydratase, and ceramide fatty acid hydroxylase (Table 4 and Table 5). This indicates that *A. sinapina* responded to betulin by prompting the cell process towards basic metabolism to produce energy (catabolism) and growth. Interestingly, a methylsterol oxidase which catalyzes the Fe-dependent first step of the removal of the two C-4 methyl groups of 4,4, dimetylzymsterol in zymosterol biosynthesis from lanosterol, ranked at position 5 of the top up-regulated transcripts (Table 4). This could indicate a feedback regulation step to process betulin. Moreover, a cytochrome P450 transcript whose activity has not been elucidated was up-regulated 9.91-fold (Table 4). Interestingly, P450 often catalyze reactions at bottleneck rate-limiting step, such as the lanosterol pathway. In addition, the conversion of betulin to betulinic aldehyde or acid, involves a redox reaction often performed by enzymes from the CYP450 family, appointing it as an interesting candidate, particularly for the biotransformation of betulin.

In the top 50 down-regulated genes from *A. sinapina* cultured cells, betulin treatment decreased the expression of transcripts involved in complex carbohydrates’ transport and degradation such as pectate lyase, hexose transporter, glycosyltransferase, permease, and lactone oxidase (Table 5). This suggests that in the presence of betulin, *A. sinapina* cells slow down processes involved in the transport and in the degradation of carbohydrates, most likely to focus on lipids’ and triterpenoids’ degradation instead. This observation might indicate a shift in *A. sinapina* catabolism from sugars to betulin as carbon source.

### 3.4. Overview of Gene Expression with Biotechnological Relevance

In the presence of betulin, a significant number of gene transcripts involved in pathways leading to terpenoid biosynthesis, enzymes catalyzing various redox reactions and survival approaches acquired by white rot fungi were differentially regulated (Table 6). As betulin is a triterpenoid retrieved in large amounts in white birch barks, special attention has been given to understand and identify enzymes involved in the triterpenoid metabolism.

### 3.5. Terpenoids

**Figure 5:**
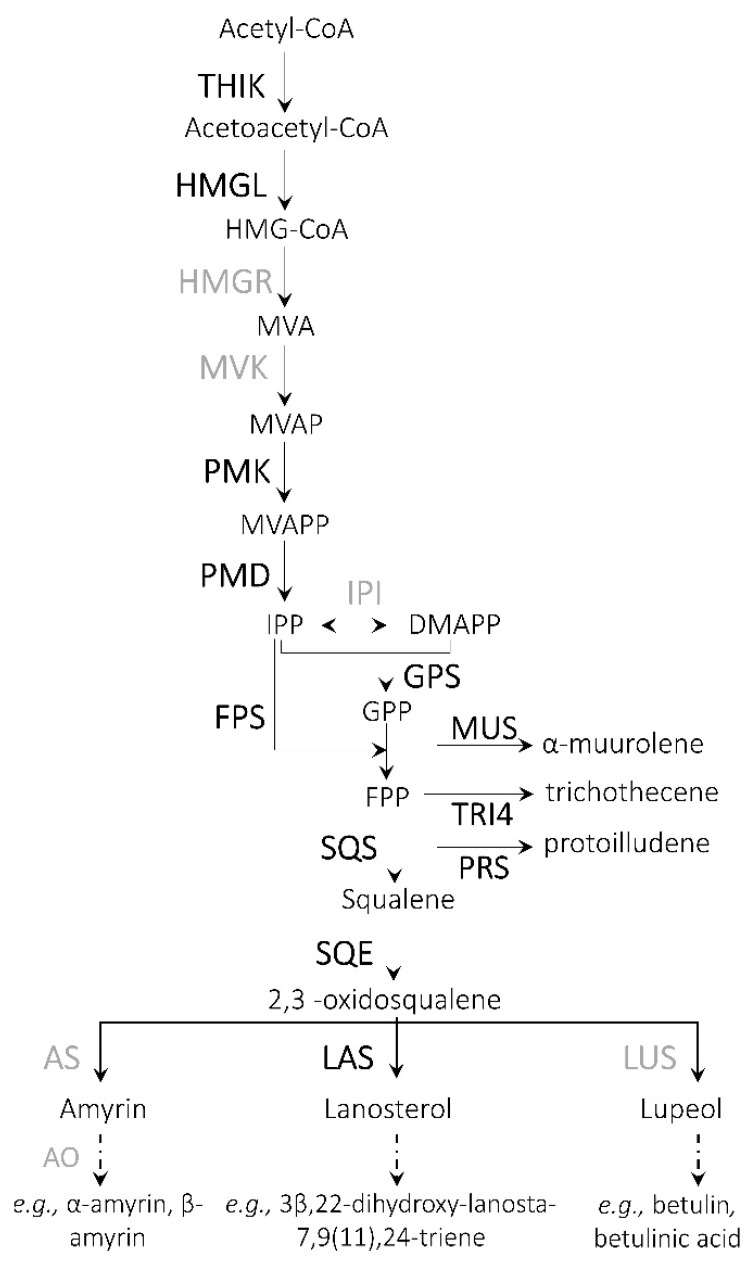
Proposed biosynthetic pathway leading to terpenoids in *A. sinapina*. Enzymes, for which corresponding gene transcripts were identified in this study, are shown in bold black, whereas the ones not found in our transcriptome are shown in bold grey. A broken arrow represents more than one biochemical reaction. Abbreviations: HMG-CoA—3-hydroxy-3-methylglutaryl-CoA; MVA—mevalonic acid; MVAP—MVA phosphate; MVAPP—MVA diphosphate; IPP—isopentenyl diphosphate; DMAPP—dimethylallyl diphosphate; GPP—geranyl diphosphate; FPP—farnesyl diphosphate; THIK—3-ketoacyl-CoA thiolase; HMGL—HMG lyase; HMGR—HMG-CoA reductase; MVK—MVA kinase; PMK—MVAP kinase; PMD—MVAPP decarboxylase; IPI—IPP isomerase; GPS—GPP synthase; FPS—FPP synthase; MUS—muurolene synthase; TRI4—trichodiene oxygenase; PRS—protoilludene synthase; SQS—squalene synthase; SQE—squalene epoxidase; AS—amyrin synthase; AO—amyrin oxidase; LAS—lanosterol synthase; LUS—lupeol synthase.

The analysis of enzymatic pathways of *A. sinapina* transcriptome with KEGG revealed a number of gene transcripts differently regulated in the presence of betulin including 23 transcripts encoding enzymes involved in terpenoid backbone biosynthesis, 3 transcripts encoding enzymes for sesquiterpenoid and triterpenoid biosynthesis, 12 transcripts for fatty acid biosynthesis and other terpenoid quinones. With regard to terpenoid backbone synthesis, genes encoding thiolase, hydroxymethylglutaryl-CoA lyase, phosphomevalonate kinase and phosphomevalonate synthase were identified. Transcripts for farnesyl diphosphate synthase, precursors of sesquiterpenoids (e.g., α-muurolene, trichothecene, protoilludene) and triterpenoids (e.g., lanosterol) have also been identified (Figure 5). Muurolene synthase, trichodiene oxygenase and protoilludene synthase for sesquiterpenoid biosynthesis were also detected as well as squalene synthase and epoxidase (Table 6).

### 3.6. Cytochrome P450s

A total of 108 transcripts encoding cytochrome P450 were detected in the transcriptome of *A. sinapina*. Among them, 32 transcripts were up-regulated in the presence of betulin (Table 6). Interestingly, eight different transcript variants of the cytochrome P450 72A14 were found up-regulated following BET treatment (Table 6). In plants, the P450 CYP716A family, and specifically CYP716A15, is implicated in the synthesis of betulinic acid [40,41]. Potential functional domains of CYP72A14, similar to plant CYP716A15, may be involved in terpenoid metabolism. Using PROSITE and SMART in silico tools [42,43], we detected that, in addition to a common P450 domain, CYP716A15 has two similar potential transmembrane domains, whereas CYP72A14 has one. Both of these domains’ sequences commonly display glycosylation, phosphorylation and N-myristoylation potential sites.

### 3.7. Enzymes Involved in Plant Cell Wall Degradation

In the pathogen–host relationship, it is important for the pathogen to use the host as a carbon source [7]. *A. sinapina* transcriptome revealed that the fungus contains key enzymes required for the degradation of plant host biomass. As such, decomposition of the plant cells require a group of enzymes that act in concert on cellulose, pectin and hemicelluloses (Table 6). Among them, gene transcripts involved in the degradation of cell walls that were highly up-regulated in *A. sinapina* transcriptome following betulin treatment included laccases, glucosidases, mannosidases, invertases, debranching enzymes, peroxidases, and glucanases (Table 6). Interestingly, two pectate lyases transcripts were down-regulated in the presence of betulin (Table 6). Moreover, as would be expected from white rot fungi, transcripts encoding cell wall degradation enzymes such as laccases, peroxidases, H_2_O_2_-generating oxidases and polysaccharide monooxygenase were present and up-regulated following the addition of betulin in *A. sinapina* transcriptome.

## 4. Discussion

Filamentous fungi are of considerable importance through their diversity, their ability to produce specialized metabolites, and their involvement in different bioprocesses. The focus of this research was on fungal phytopathogen *A. sinapina* and its possible application in various bioprocesses such as betulin biotransformation.

We obtained comprehensive transcriptome data from *A. sinapina* by analyzing transcripts from samples treated or not treated with betulin using Illumina sequencing and Trinity de novo transcriptome assembly. Using a similar method, A. L. Ross-Davis (2013) assembled and analyzed the transcriptome of *A. solidipes* [7]. Compared to their work, the average lengths and N50 of our contigs, scaffolding and unigenes were longer. In another research work on the saprophytic fungus *Wolfiporia cocos*, Illumina sequencing of the transcriptome yielded a total of 38,722,186 reads, which were assembled into 60,354 contigs with N50 of 765 bp. Here, we obtained 4–7 times more reads, ensuring a broad coverage and a more contiguous assembly, as confirmed by the average transcript length of 2051 bp. The average length of eukaryotic transcript genes is greater than 1 kb (1135–1695 bp in yeast, 1108–2667 bp in human) 103,267 (37.75%), 61,272 (23.53%), 105,195 (40.39%), 45,683 (16.70%) unigenes had similar sequences with the GO, COG, Pfam and KEGG databases, respectively. The most abundant GO categories and functional groups here (Figure 2) were consistent with those of the transcriptomic analysis of *A. solidipes* [7]. In addition, 128,370 of our transcripts showed no similarity with known sequences based on available databases until now. Hence, we have discovered new genes from *A. sinapina* that had not been previously identified by other genomics researchers. The KEGG annotation indicated that the three most abundant categories were the metabolic pathways, biosynthesis of specialized metabolites, biosynthesis of antibiotics and carbon metabolism.

Treatment of *A. sinapina* cell culture with betulin revealed differential regulation of a large number of gene transcripts putatively involved in the development of white rot disease. Interestingly, 3863 transcripts were up-regulated, and 80 down-regulated in the presence of betulin. Transcripts of enzymes, such as cytochrome P450, lanosterol synthase, glycoside hydrolase, 3-ketoacyl-CoA thiolase 5 and C4-methyl sterol oxidase involved in the specialized metabolites’ biosynthesis were retrieved in the top 50 up-regulated genes. P450s play essential roles in biological pathways, such as the biosynthesis of terpenoids and alkaloids. Because of their ability to introduce oxygen into non-activated C-H bonds, P450s are attractive tools for biotechnology applications such as biotransformation methods as an alternative to classical chemical synthesis, particularly in the field of triterpenoid oxidation [44]. Here, the CYP72A sub-family was found to be up-regulated in the presence of betulin. In plants, CYP72A is required for the metabolism of specialized triterpenoids that carry out species-specific functions, such as chemical defense response to specialized pathogens [45,46]. To date, no study has been published on the possible function of this sub-family and very little is known on P450 in general in fungi [44]. P450 enzymes often metabolize toxic substances into more polar and water-soluble compounds, and therefore, play an important role in detoxification, which may explain why they were up-regulated in the transcriptome of *A. sinapina* in the presence of antimicrobial betulin [47]. In addition, a number of gene transcripts of the P450 family identified in the transcriptome of *A. sinapina* and up-regulated by betulin treatment have not yet been functionally defined.

We also identified numerous transcripts encoding enzymes involved in terpenoids biosynthesis and proposed a reconstructed pathway. Interestingly, despite some differences (i.e., lanosterol synthase), this biosynthetic pathway of *A. sinapina* is very similar to the one present in plants. Thus, fungi, and probably plant pathogen ones, may have evolved to produce plant-like enzymes that enable them to better benefit from host’s metabolites. In recent years, Basidiomycota white rot fungi have demonstrated their ability to degrade all components of lignocellulosic biomass, and have therefore, become promising candidates for biotechnological applications, such as the pretreatment of plant feedstocks for conversion to bioethanol, other biofuels and the production of value-added bioproducts. Genomic and transcriptomic studies of several fungi of the Basidiomycota Agaricomycetes family have revealed genes encoding enzymes that cause wood degradation [9]. Oxidation and depolymerization of lignin are considered a significant barrier to the industrial use of lignocelluloses from multiple sources such as wood residues. The lignin may be modified, and sometimes mineralized by extracellular oxidoreductases produced by white rot fungi, including lignin and manganese peroxidases, in conjunction with some enzymes generating hydrogen peroxide such as glyoxal oxidases or aryl alcohol oxidases [9]. The versatility of North species such as *A. sinapina* to biodegrade all structural components of plant cell walls is clearly shown in its transcriptome, which has a large number of genes associated to the degradation of plant cell walls, including enzymes that degrade lignin, cellulose, hemicellulose, and pectin [48]. Laccases, different peroxidases, several H_2_O_2_-generating oxidases and polysaccharide monooxygenase were present and up-regulated by the addition of betulin in *A. sinapina* transcriptome. Several of these enzyme types have been reported in other white rot fungi [49]. For example, a manganese-peroxidase was found in the fungal strain of *C. subvermisposa* with more than 11 isoforms [50].

Laccases are described in various organisms, from bacteria to insects, and have the ability to oxidize various phenolic substrates by reducing the oxygen in water [50]. Recently, a growing interest for polysaccharide mono-oxygenases uncovered in large numbers in gene analyses of white rot fungi has been shown [51]. They are a class of copper-dependent enzymes that play a role in boosting the degradation of polysaccharides in lignocellulosic biomasses. They are capable of weak hydrolysis of beta-glucan, carboxymethylcellulose, lichenin, wheat arabinoxylan and birch xylan. In addition, polysaccharide monooxygenases in conjunction with other cellulolytic enzymes stimulate the hydrolysis of lignocellulosic substrates, such as hydrothermally pretreated wheat straw [51,52]. Here, Cel16 was up-regulated in the presence of betulin.

The potential of *A. sinapina* in biomass deconstruction is also shown by the detection of various transcripts encoding proteins belonging to the glycosylhydrolase family, i.e., beta-glucosidase, alpha-xylosidase, probable exo-1.4 beta xylosidase, endo-1,4-beta-xylanase, bifunctional xylanase/deacetylase, xyloglucanase, polygalacturonase and exopolygalacturonase, endo-beta-1,4-glucanase D, alpha-xylosidase and alpha-glucosidase, arabinan endo-1,5-alpha-L-arabinosidase A and beta-mannosidase

Regarding enzymes involved in cell wall modifications, it is noteworthy that there were more gene transcripts up-regulated than down-regulated. Only pectate lyases (PL) were down-regulated. The cell wall is an important barrier against pathogens that attack plants. Pathogens produce pectinases, polygalacturonases, pectins, PL and pectin esterases to digest homogalacturonans and rhamnogalacturonans [53]. In 2006 and 2013, studies indicated that secretion of PL and fungal pathogenicity could be influenced by nutritional factors, such as the presence of nitrogen in culturing medium. [54,55]. In addition, PL have an absolute requirement for Ca^2+^ ions [56]. The negative regulation of PL could be explained by different factors, such as deficiencies in nitrogen or Ca^2+^ in the culture medium, pH, temperature, and finally, by the presence of triterpenoids (i.e., betulin), which could be inhibitors to this enzyme [57].

## 5. Conclusions

In this study, we used *A. sinapina*, a root disease pathogen of hardwood hosts as a model to explore de novo assembly of a complete transcriptome and to assess changes in transcripts levels following betulin treatment using the Illumina paired-end sequencing method. The quality and the depth of our transcriptomic assembly is significantly improved, compared to other fungal transcriptome studies, and will serve as a basis for further transcriptomic research on North American Armillaria species. A total of 343,289 unigenes were obtained from *A. sinapina* mycelium following Trinity de novo assembly. Various gene transcripts orthologs, with important roles in the degradation of plant cell walls and involved in the production of metabolites, were identified and annotated. These results demonstrate that Illumina paired-end sequencing is a fast and cost-effective approach for the discovery of new genes in a non-model organism. This research may help to optimize applications exploiting *A. sinapina* and will facilitate the identification of candidate genes for uses in biotransformation, and also as an enzyme platform for the degradation and depolymerization of woody biomass to produce biofuels and a chemical product. Further work on the nature of the genes of the cytochrome P450 family and the non-annotated genes could reveal key elements in the relationship between *A. sinapina* and its plant host, and thus, its involvement in the life cycle of the forest ecosystem.

## Figures and Tables

**Table 1:** Result of Illumina sequencing output and assembly for *Armillaria sinapina.*

**Read Trimming and Clipping of Adapters**	
Raw paired reads ^a^	170,592,464
Surviving paired reads ^b^	166,006,772
Surviving paired reads (%) ^c^	97.31
**Trinity *De Novo Assembly***	
Nb. Transcripts^d^	273,561
Nb. Components^d^	99,358
Total Transcripts Length (bp)	561,145,023
Max. Transcript Length (bp)	16,405
Min. Transcript Length (bp)	201
Median Transcript Length (bp)	1456
Mean Transcript Length (bp)	2051
N50 (bp)	3523
**BLAST Annotation and Filtered Annotated Components**	
Nb. Transcripts	121,959
Nb. Components	13,544
Total Transcripts Length (bp)	288,576,284
Max. Transcript Length (bp)	16,405
Min. Transcript Length (bp)	298
Median Transcript Length (bp)	3661
Mean Transcript Length (bp),	4033
N50 (bp)	5081

^a^ Number of Paired Reads obtained from the sequencer, ^b^ Number of Remaining Paired Reads after the trimming step, ^c^ Percentage of Surviving Paired Reads/Raw Paired Reads, ^d^ Trinity has created a list of transcripts (contigs) representing the transcriptome isoforms which are grouped in components loosely representing genes. Transcript names are prefixed by the component/gene name e.g., transcripts c115_g5_i1 and c115_g5_i2 are derived from the same isolated de Bruijn graph, and therefore, share the same component/gene number c115_g5.

**Table 2:** Summary of the functional annotation of transcript sequences from *A. sinapina* obtained from public databases.

Public Database	Number of Transcripts	Percentage (%)
Blast-GO annotations	103,267	37.75
COG annotations	61,272	23.53
Pfam annotations	45,683	16.69
KEGG annotations	105,195	40.39
Total number of sequences not annotated	164,622	60.18
Total number of sequences annotated	108,939	39.82
Total of transcript sequences	273,561	100.00

**Table 3:** The number of differentially expressed transcripts between the different databases.

Set	Differentially Expressed Transcripts	Up-Regulated	Down-Regulated
BET vs. Control	3943	3863	80

**Table 4:** 50 up-regulated transcripts of *A. sinapina* in cells cultivated in the presence of betulin.

Rank	Log2 FC	Description	Species	E-Value	Accession
1	10.96	3-ketoacyl-CoA thiolase 5	*Grifola fondosa*	0	OBZ79350.1
2	10.92	NAD-P-binding protein	*Trametes versicolor*	0	XP_008044855.1
3	10.82	Hypothetical protein	*Trametes versicolor*	0	XP_008042994.1
4	10.73	No hit			
5	10.68	C4-methyl sterol oxidase	*Trametes versicolor*	0	XP_008044057.1
6	10.64	Golgi apparatus membrane protein TVP38	*Trametes pubescens*	0	OJT05825.1
7	10.56	Hypothetical protein	*Trametes versicolor*	0	XP_008035434.1
8	10.55	Hypothetical protein	*Trametes versicolor*	0	XP_008034798.1
9	10.51	HSP20-like chaperone	*Trametes versicolor*	0	XP_008040595.1
10	10.51	NADPH oxidase isoform 2	*Trametes versicolor*	0	XP_008043698.1
11	10.44	GroES-like protein	*Trametes versicolor*	0	XP_008042467.1
12	10.44	Heat shock protein	*Trametes versicolor*	0	XP_008036836.1
13	10.33	Phosphoglycerate kinase	*Trametes versicolor*	0	XP_008036919.1
14	10.32	Heat shock protein 70	*Trametes versicolor*	0	XP_008040622.1
15	10.31	Hypothetical protein	*Trametes versicolor*	0	XP_008034620.1
16	10.29	NAD-P-binding protein	*Trametes versicolor*	0	XP_008044855.1
17	10.28	Hypothetical protein	*Trametes versicolor*	0	XP_008042390.1
18	10.27	Hypothetical protein	*Trametes versicolor*	0	XP_008042803.1
19	10.25	Hypothetical protein	*Trametes versicolor*	0	XP_008041395.1
20	10.23	Calnexin-like protein	*Trametes pubescens*	0	OJT08454.1
21	10.21	No hit			
22	10.21	Heat shock protein 30	*Trametes versicolor*	0	XP_008045263.1
23	10.20	Pyruvate decarboxylase	*Trametes versicolor*	0	XP_008037637.1
24	10.17	No hit			
25	10.15	Hypothetical protein	*Trametes versicolor*	0	XP_008037020.1
26	10.08	Hypothetical protein	*Trametes versicolor*	0	XP_008045579.1
27	10.08	Fumarate reductase	*Trametes pubescens*	0	OJT15326.1
28	10.06	Acid protease	*Trametes versicolor*	0	XP_008034004.1
29	10.06	No hit			
30	10.06	Glycoside hydrolase	*Trametes versicolor*	0	XP_008038270.1
31	10.06	Hypothetical protein	*Trametes versicolor*	0	XP_008035168.1
32	10.05	No hit			
33	10.04	No hit			
34	10.01	Phenylalanine-t-RNA synthethase	*Trametes versicolor*	0	XP_008035697.1
35	10.01	No hit			
36	9.99	No hit			
37	9.98	Hypothetical protein	*Trametes versicolor*	0	XP_008045519.1
38	9.96	Hypothetical protein	*Trametes versicolor*	0	XP_008040976.1
39	9.96	No hit			
40	9.94	Hypothetical protein	*Trametes pubescens*	0	OJT02899.1
41	9.93	Aspartic peptidase A1	*Trametes versicolor*	0	XP_008035722.1
42	9.93	Heat shock protein 20-like chaperone	*Trametes versicolor*	0	XP_008037816.1
43	9.91	Cytochrome P450	*Trametes versicolor*	0	XP_008033952.1
44	9.91	Hypothetical protein	*Trametes versicolor*	0	XP_008035218.1
45	9.88	Delta 9-fatty acid desaturase	*Trametes versicolor*	0	XP_008041237.1
46	9.88	No hit			
47	9.88	Phosphopyruvate hydratase	*Trametes versicolor*	0	XP_008032334.1
48	9.88	Ceramide very long chain fatty acid hydroxylase	*Trametes pubescens*	0	OJT03476.1
49	9.86	No hit			
50	9.86	No hit			

**Table 5:** Top 50 down-regulated transcripts of *A. sinapina* in cells cultivated in the presence of betulin.

Rank	Log2 FC	Description	Species	E-Value	Accession
1	−6.26	AFG1-like ATPase	*Armillaria gallica*	0	PBL02956.1
2	−6.23	Pectate lyase	*Armillaria gallica*	0	PBL03123.1
3	−5.82	Putative O-fucosyltransferease-like protein	* Moniliophtora roreri *	1 E-124	KTB33430.1
4	−5.45	Hypothetical protein	*Armillaria solidipes*	9 E-84	PBK63479.1
5	−5.38	Uncharacterized protein	*Armillaria ostoyae*	1 E-147	SJL03005.1
6	−5.32	Uncharacterized protein	*Armillaria ostoyae*	1 E-146	SJL03005.1
7	−5.08	Hexose transporter	*Armillaria gallica*	0	PBK93947.1
8	−4.88	Glycosyltransferase	*Armillaria solidipes*	0	PBK58814.1
9	−4.74	Uncharacterized protein	*Armillaria ostoyae*	0	SJL10357.1
10	−4.65	Casein kinase II beta subunit	*Armillaria gallica*	0	PBL02672.1
11	−3.47	Hypothetical protein	*Armillaria gallica*	0	PBL00259.1
12	−3.42	No hit			
13	−3.36	Heavy metal translocatin	*Armillaria solidipes*	0	PBK63629.1
14	−3.31	Hypothetical protein	*Armillaria solidipes*	0	PBK76216.1
15	−3.25	WD40 repeat-like protein	*Armillaria solidipes*	5 E-168	PBK69270.1
16	−3.16	Kinase-like protein	*Armillaria solidipes*	4 E-129	PBK63656.1
17	−3.02	Hypothetical protein	*Armillaria gallica*	0	PBK93574.1
18	−2.97	Nicotinamide mononucleotide related permease	*Armillaria ostoyae*	0	SJL10502.1
19	−2.97	Alpha-aminoadipate reductase Lys1p	*Armillaria gallica*	0	PBK98805.1
20	−2.91	Hypothetical protein	*Armillaria solidipes*	9 E-64	PBK75291.1
21	−2.84	Hypothetical protein	*Armillaria solidipes*	0	PBK69763.1
22	−2.66	Cytochrome P450	*Armillaria gallica*	2 E-133	PBK89827.1
23	−2.63	Kinase-like protein	*Armillaria solidipes*	0	PBK71971.1
24	−2.59	Hypothetical protein	*Armillaria gallica*	0	PBL01386.1
25	−2.39	Uncharacterized protein	*Armillaria ostoyae*	0	SJL04719.1
26	−2.37	Hypothetical protein	*Armillaria gallica*	0	PBK93754.1
27	−2.26	No hit			
28	−2.23	NAD-dependent deacylase	*Armillaria ostoyae*	2 E-90	SJL08753.1
29	−2.20	No hit			
30	−2.16	No hit			
31	−2.12	Hypothetical protein	*Armillaria gallica*	3 E-70	PBK96234.1
32	−2.11	Uncharacterized protein	*Armillaria ostoyae*	1 E-149	SJL09942.1
33	−2.08	No hit			
34	−2.02	No hit			
35	−2.01	No hit			
36	−1.99	No hit			
37	−1.96	HOOK-domain-containing protein	*Armillaria solidipes*	0	PBK77734.1
38	−1.94	DNA excision repair protein	*Hypsizygus marmoreus*	0	RDB19634.1
39	−1.94	Uncharacterized protein	*Armillaria ostoyae*	0	SJK99761.1
40	−1.81	BRO1-domain-containing protein	*Armillaria gallica*	0	PBK82405.1
41	−1.78	Ribonuclease subunit	*Armillaria ostoyae*	0	SJL08201.1
42	−1.75	Hypothetical protein	*Armillaria solidipes*	0	PBK63259.1
43	−1.67	No hit			
44	−1.67	Uncharacterized protein	*Armillaria ostoyae*	0	SJL12868.1
45	−1.64	BRO1-domain-containing protein	*Armillaria gallica*	0	PBK82405.1
46	−1.62	Uncharacterized protein	*Armillaria ostoyae*	0	SJL12868.1
47	−1.61	Uncharacterized protein	*Armillaria ostoyae*	0	SJL09034.1
48	−1.60	L-gulonolactone D-arabinono-1,4-lactone oxidase	*Armillaria solidipes*	0	PBK71727.1
49	−1.59	Vacuole import and degradation VID27	*Armillaria solidipes*	0	PBK69736.1
50	−1.58	Uncharacterized protein	*Armillaria ostoyae*	0	SJL09949.1

**Table 6:** Differently expressed biotechnologically-relevant transcripts of *A. sinapina* cells in the presence of betulin.

Transcript Name	Log2FC	Annotation
***Terpenoids***		
Armillaria_TRINITY_DN29150_c0_g1_i1	10.68	Methylsterol monooxygenase
Armillaria_TRINITY_DN22917_c0_g1_i3	9.00	Lanosterol synthase
Armillaria_TRINITY_DN3215_c0_g1_i1	8.85	C-5 sterol desaturase
Armillaria_TRINITY_DN31064_c0_g1_i13	8.70	Lanosterol 14-alpha demethylase
Armillaria_TRINITY_DN6566_c0_g1_i2	8.27	Sterol 24-C-methyltransferase
Armillaria_TRINITY_DN34616_c0_g1_i1	7.28	Alpha-muurolene synthase
Armillaria_TRINITY_DN17473_c0_g1_i1	7.22	Lanosterol 14-alpha demethylase
Armillaria_TRINITY_DN45244_c0_g1_i1	7.17	Squalene epoxidase
Armillaria_TRINITY_DN18035_c0_g1_i1	7.13	Delta(14)-sterol reductase
Armillaria_TRINITY_DN45179_c0_g1_i1	6.96	Squalene synthase
Armillaria_TRINITY_DN54064_c0_g1_i1	6.94	Sterol-4-alpha-carboxylate 3-dehydrogenase
Armillaria_TRINITY_DN13258_c0_g1_i1	6.93	Sterol 14-demethylase
Armillaria_TRINITY_DN12757_c0_g1_i3	6.91	Alpha-muurolene synthase
Armillaria_TRINITY_DN10601_c0_g1_i1	6.78	Farnesyltransferase/geranylgeranyltransferase subunit alpha
Armillaria_TRINITY_DN10022_c0_g1_i1	6.78	Isopentenyl-diphosphate Delta-isomerase
Armillaria_TRINITY_DN4217_c0_g1_i1	6.75	Delta(24(24(1)))-sterol reductase
Armillaria_TRINITY_DN55954_c0_g1_i1	6.71	C-8 sterol isomerase
Armillaria_TRINITY_DN9929_c0_g1_i1	6.66	Diphosphomevalonate decarboxylase
***CYP450s***		
Armillaria_TRINITY_DN20121_c0_g1_i1	9.91	Cytochrome P450 72A14
Armillaria_TRINITY_DN8574_c0_g1_i1	8.67	Cytochrome P450 72A14
Armillaria_TRINITY_DN61797_c0_g1_i1	8.50	Cytochrome P450 4F5
Armillaria_TRINITY_DN5903_c0_g1_i1	8.28	Cytochrome P450 67
Armillaria_TRINITY_DN4166_c0_g1_i1	7.74	Cytochrome P450 61
Armillaria_TRINITY_DN31064_c0_g1_i10	7.60	Cytochrome P450 72A14
Armillaria_TRINITY_DN23798_c1_g2_i3	7.44	Docosahexaenoic acid omega-hydroxylase CYP4F3
Armillaria_TRINITY_DN31064_c0_g1_i5	7.21	Cytochrome P450 72A14
Armillaria_TRINITY_DN23798_c1_g2_i1	7.16	Docosahexaenoic acid omega-hydroxylase CYP4F3
Armillaria_TRINITY_DN9123_c0_g1_i1	7.14	Cytochrome P450 4F22
Armillaria_TRINITY_DN23798_c1_g1_i1	7.00	Cytochrome P450 3A9
Armillaria_TRINITY_DN56547_c0_g1_i1	6.97	Putative cytochrome P450 CYP13A10
Armillaria_TRINITY_DN153_c0_g1_i1	6.95	Cytochrome P450 3A24
Armillaria_TRINITY_DN13346_c0_g1_i1	6.95	Cytochrome P450 52E1
Armillaria_TRINITY_DN7413_c0_g2_i1	6.90	Cytochrome P450 3A24
Armillaria_TRINITY_DN9340_c0_g1_i1	6.89	Cytochrome P450 4A4
Armillaria_TRINITY_DN31064_c0_g1_i27	6.89	Cytochrome P450 72A14
Armillaria_TRINITY_DN14362_c0_g1_i1	6.86	Taurochenodeoxycholic 6 alpha-hydroxylase
Armillaria_TRINITY_DN21389_c0_g1_i1	6.85	Docosahexaenoic acid omega-hydroxylase CYP4F3
Armillaria_TRINITY_DN31064_c0_g1_i3	6.71	Cytochrome P450 72A14
Armillaria_TRINITY_DN6971_c0_g1_i1	6.65	Putative cytochrome P450 CYP13A8
Armillaria_TRINITY_DN3618_c0_g1_i1	6.58	Cytochrome P450 72A15
Armillaria_TRINITY_DN38009_c0_g1_i1	6.57	Cytochrome P450 72A14
Armillaria_TRINITY_DN31064_c0_g1_i21	6.50	Cytochrome P450 72A14
Armillaria_TRINITY_DN30372_c0_g1_i50	4.07	Cytochrome P450 98A1
Armillaria_TRINITY_DN32870_c0_g1_i19	3.84	Fumitremorgin C synthase
Armillaria_TRINITY_DN32870_c0_g1_i5	3.63	O-methylsterigmatocystin oxidoreductase
Armillaria_TRINITY_DN32011_c0_g1_i15	1.69	Docosahexaenoic acid omega-hydroxylase CYP4F3
Armillaria_TRINITY_DN33108_c1_g2_i4	1.55	Leukotriene-B4 omega-hydroxylase 3
Armillaria_TRINITY_DN31587_c1_g1_i22	1.02	Cytochrome P450 4F1
Armillaria_TRINITY_DN33118_c0_g1_i13	0.99	Cytochrome P450 52A6
Armillaria_TRINITY_DN33118_c0_g1_i20	0.93	Cytochrome P450 52A6
***Cell Wall Enzymes***		
Armillaria_TRINITY_DN19168_c0_g1_i1	9.19	Probable feruloyl esterase A
Armillaria_TRINITY_DN31438_c0_g4_i1	8.93	Laccase
Armillaria_TRINITY_DN22833_c0_g1_i1	8.87	Glucan 1,3-beta-glucosidase D
Armillaria_TRINITY_DN20665_c0_g1_i1	8.76	Beta-mannosidase A
Armillaria_TRINITY_DN8404_c0_g1_i1	8.75	Probable glucan 1,3-beta-glucosidase D
Armillaria_TRINITY_DN21709_c0_g1_i2	8.68	Polysaccharide monooxygenase
Armillaria_TRINITY_DN44356_c0_g1_i1	7.99	Mannosyl-oligosaccharide 1,2-alpha-mannosidase
Armillaria_TRINITY_DN15187_c0_g1_i1	7.52	Glucosidase 2 subunit beta
Armillaria_TRINITY_DN2359_c0_g1_i1	7.42	Invertase 2
Armillaria_TRINITY_DN20388_c0_g1_i4	7.17	Probable feruloyl esterase B-1
Armillaria_TRINITY_DN31438_c0_g6_i2	7.09	Laccase-1
Armillaria_TRINITY_DN13563_c0_g1_i2	7.06	Probable glucan endo-1,3-beta-glucosidase
Armillaria_TRINITY_DN16550_c0_g1_i1	7.05	Glucosidase 2 subunit alpha
Armillaria_TRINITY_DN43871_c0_g1_i1	7.04	Glucan endo-1,3-alpha-glucosidase agn1
Armillaria_TRINITY_DN3408_c0_g1_i1	7.04	Glycogen debranching enzyme
Armillaria_TRINITY_DN31438_c0_g3_i1	6.97	Laccase-2
Armillaria_TRINITY_DN71958_c0_g1_i1	6.94	Probable glucan 1,3-beta-glucosidase D
Armillaria_TRINITY_DN11046_c0_g1_i2	6.91	Glucan 1,3-beta-glucosidase
Armillaria_TRINITY_DN955_c0_g1_i1	6.83	Alpha-mannosidase
Armillaria_TRINITY_DN55139_c0_g1_i1	6.82	Probable alpha/beta-glucosidase
Armillaria_TRINITY_DN24913_c0_g1_i1	6.81	Mannosyl-oligosaccharide alpha-1,2-mannosidase
Armillaria_TRINITY_DN33885_c0_g1_i1	6.78	Mannosyl-oligosaccharide 1,2-alpha-mannosidase
Armillaria_TRINITY_DN11477_c0_g1_i1	6.68	Glucan 1,3-beta-glucosidase
Armillaria_TRINITY_DN38622_c0_g1_i1	6.63	Uncharacterized family 31 glucosidase
Armillaria_TRINITY_DN12447_c0_g1_i1	6.55	Probable endo-beta-1,4-glucanase D
Armillaria_TRINITY_DN13227_c0_g2_i1	6.55	Probable glucan 1,3-beta-glucosidase D
Armillaria_TRINITY_DN13426_c0_g1_i1	6.55	Versatile peroxidase VPL1
Armillaria_TRINITY_DN13563_c0_g1_i1	6.54	Probable glucan endo-1,3-beta-glucosidase
Armillaria_TRINITY_DN12447_c0_g1_i2	6.53	Probable endo-beta-1,4-glucanase D
Armillaria_TRINITY_DN15833_c0_g1_i1	6.49	Probable beta-glucosidase A
Armillaria_TRINITY_DN32420_c0_g1_i1	2.03	Xyloglucanase
Armillaria_TRINITY_DN31740_c0_g1_i23	−5.82	Pectate lyase A
Armillaria_TRINITY_DN31740_c0_g1_i15	−6.23	Pectate lyase A

## References

[1] Stewart J.E., Kim M.-S., Klopfenstein N.B. (2018). Molecular Genetic Approaches Toward Understanding Forest-Associated Fungi and Their Interactive Roles Within Forest Ecosystems. Curr. For. Rep..

[2] Araujo R., Sampaio-Maia B. (2018). Fungal Genomes and Genotyping. Advances in Applied Microbiology.

[3] Laperriere G., Desgagne-Penix I., Germain H. (2018). DNA distribution pattern and metabolite profile of wild edible lobster mushroom (Hypomyces lactifluorum/Russula brevipes). Genome Natl. Res. Counc. Can. Genome Cons. Natl. Rech. Can..

[4] Genevieve L., Pierre-Luc C., Roxanne G.-T., Amélie M., Danny B., Vincent M., Hugo G. (2019). Estimation of Fungal Diversity and Identification of Major Abiotic Drivers Influencing Fungal Richness and Communities in Northern Temperate and Boreal Quebec Forests. Forests.

[5] Sipos G., Anderson J.B., Nagy L.G. (2018). Armillaria. Curr. Biol..

[6] Heinzelmann R., Dutech C., Tsykun T., Labbé F., Soularue J.-P., Prospero S. (2019). Latest advances and future perspectives in Armillaria research. Can. J. Plant Pathol..

[7] Ross-Davis A., Stewart J., Hanna J., Kim M.S., Knaus B., Cronn R., Rai H., Richardson B., McDonald G., Klopfenstein N. (2013). Transcriptome of an A rmillaria root disease pathogen reveals candidate genes involved in host substrate utilization at the host–pathogen interface. For. Pathol..

[8] Schwartz M., Perrot T., Aubert E., Dumarçay S., Favier F., Gérardin P., Morel-Rouhier M., Mulliert G., Saiag F., Didierjean C. (2018). Molecular recognition of wood polyphenols by phase II detoxification enzymes of the white rot Trametes versicolor. Sci. Rep..

[9] Mäkinen M.A., Risulainen N., Mattila H., Lundell T.K. (2018). Transcription of lignocellulose-decomposition associated genes, enzyme activities and production of ethanol upon bioconversion of waste substrate by Phlebia radiata. Appl. Microbiol. Biotechnol..

[10] Aranda E. (2016). Promising approaches towards biotransformation of polycyclic aromatic hydrocarbons with Ascomycota fungi. Curr. Opin. Biotechnol..

[11] Bérubé J., Dessureault M. (1988). Morphological characterization of Armillaria ostoyae and Armillaria sinapina sp. nov. Can. J. Bot..

[12] Klopfenstein N.B., Stewart J.E., Ota Y., Hanna J.W., Richardson B.A., Ross-Davis A.L., Elías-Román R.D., Korhonen K., Keča N., Iturritxa E. (2017). Insights into the phylogeny of Northern Hemisphere Armillaria: Neighbor-net and Bayesian analyses of translation elongation factor 1-α gene sequences. Mycologia.

[13] McLaughlin J. (2001). Distribution, hosts, and site relationships of Armillaria spp. in central and southern Ontario. Can. J. For. Res..

[14] Mallett K. (1990). Host range and geographic distribution of Armillaria root rot pathogens in the Canadian prairie provinces. Can. J. For. Res..

[15] Dehelean C.A., Şoica C., Ledeţi I., Aluaş M., Zupko I., Gǎluşcan A., Cinta-Pinzaru S., Munteanu M. (2012). Study of the betulin enriched birch bark extracts effects on human carcinoma cells and ear inflammation. Chem. Cent. J..

[16] Joseph A., Kutty N.G., Moorkoth S., Alex A.T. (2019). In vitro and In vivo Anticancer Activity of Semisynthetic Derivatives of Betulinic acid. Lat. Am. J. Pharm..

[17] Wang X., Yuan Z., Zhu L., Yi X., Ou Z., Li R., Tan Z., Pozniak B., Obminska-Mrukowicz B., Wu J. (2019). Protective Effects of Betulinic Acid on Intestinal Mucosal Injury Induced by Cyclophosphamide in Mice. Pharmacol. Rep..

[18] Huang L., Li J., Ye H., Li C., Wang H., Liu B., Zhang Y. (2012). Molecular characterization of the pentacyclic triterpenoid biosynthetic pathway in Catharanthus roseus. Planta.

[19] Fukushima E.O., Seki H., Ohyama K., Ono E., Umemoto N., Mizutani M., Saito K., Muranaka T. (2011). CYP716A Subfamily Members are Multifunctional Oxidases in Triterpenoid Biosynthesis. Plant Cell Physiol..

[20] Liu J., Fu M.L., Chen Q.H. (2011). Biotransformation optimization of betulin into betulinic acid production catalysed by cultured Armillaria luteo-virens Sacc ZJUQH100-6 cells. J. Appl. Microbiol..

[21] Yin J., Ma H., Gong Y., Xiao J., Jiang L., Zhan Y., Li C., Ren C., Yang Y. (2013). Effect of MeJA and Light on the Accumulation of Betulin and Oleanolic Acid in the Saplings of White Birch (*Betula platyphylla* Suk.). Am. J. Plant Sci..

[22] Mandlik A., Livny J., Robins W.P., Ritchie J.M., Mekalanos J.J., Waldor M.K. (2011). RNA-Seq-based monitoring of infection-linked changes in Vibrio cholerae gene expression. Cell Host Microbe.

[23] Cao S., Zhu L., Nie H., Yin M., Liu G., Yan X. (2018). De novo assembly, gene annotation, and marker development using Illumina paired-end transcriptome sequencing in the Crassadoma gigantea. Gene.

[24] Zhan M., Tian M., Wang W., Li G., Lu X., Cai G., Yang H., Du G., Huang L. (2020). Draft genomic sequence of Armillaria gallica 012m: Insights into its symbiotic relationship with Gastrodia elata. Braz. J. Microbiol..

[25] Collins C. (2013). A Genomic and Proteomic Investigation of the Plant Pathogen Armillaria mellea: Buried Treasure or Hidden Threat?. Ph.D. Thesis.

[26] Sipos G., Qi W., Künzli M., Okoniewski M., Rigling D. 454 sequencing of transcriptomes for virulent and non-virulent Armillaria ostoyae strains and identification of their secretomes. Proceedings of the XIII Conference”Root and Butt Rot of Forest Trees” IUFRO Working Party 7.02.01.

[27] Ross-Davis A.L., Stewart J.E., Hanna J.W., Kim M.-S., Cronn R.C., Rai H.S., Richardson B.A., McDonald G.I., Klopfenstein N.B., Zeglen S., Palacios P. (2011). De novo assembly and transcriptome characterization of an Armillaria solidipes mycelial fan. Proceedings of the 59th Annual Western International Forest Disease Work Conference.

[28] Liu M.-M., Xing Y.-M., Zhang D.-W., Guo S.-X. (2015). Transcriptome analysis of genes involved in defence response in Polyporus umbellatus with Armillaria mellea infection. Sci. Rep..

[29] Engels B., Heinig U., Grothe T., Stadler M., Jennewein S. (2011). Cloning and characterization of an *Armillaria gallica* cDNA encoding protoilludene synthase, which catalyzes the first committed step in the synthesis of antimicrobial melleolides. J. Biol. Chem..

[30] Ming-liang F., Jing L., Ya-chen D., Yu F., Ruo-si F., Qi-he C., Xiao-jie L. (2011). Effect of ionic liquid-containing system on betulinic acid production from betulin biotransformation by cultured Armillaria luteo-virens Sacc cells. Eur. Food Res. Technol..

[31] Zhang H.-F., He G.-Q., Liu J., Ruan H., Chen Q.-H., Zhang Q., Wang J.-L., Zhang H.-B. (2008). Production of gastrodin through biotransformation of p-2-hydroxybenzyl alcohol by cultured cells of Armillaria luteo-virens Sacc. Enzyme Microbial Technol..

[32] Fradj N., Gonçalves dos Santos K.C., de Montigny N., Awwad F., Boumghar Y., Germain H., Desgagné-Penix I. (2019). RNA-Seq de Novo Assembly and Differential Transcriptome Analysis of Chaga *(Inonotus obliquus)* Cultured with Different Betulin Sources and the Regulation of Genes Involved in Terpenoid Biosynthesis. Int. J. Mol. Sci..

[33] Bolger A.M., Lohse M., Usadel B. (2014). Trimmomatic: A flexible trimmer for Illumina sequence data. Bioinformatics.

[34] Brown C.T., Howe A., Zhang Q., Pyrkosz A.B., Brom T.H. (2012). A reference-free algorithm for computational normalization of shotgun sequencing data. arXiv.

[35] Grabherr M.G., Haas B.J., Yassour M., Levin J.Z., Thompson D.A., Amit I., Adiconis X., Fan L., Raychowdhury R., Zeng Q. (2011). Full-length transcriptome assembly from RNA-Seq data without a reference genome. Nat. Biotechnol..

[36] Langmead B., Trapnell C., Pop M., Salzberg S.L. (2009). Ultrafast and memory-efficient alignment of short DNA sequences to the human genome. Genome Biol..

[37] Li B., Dewey C.N. (2011). RSEM: Accurate transcript quantification from RNA-Seq data with or without a reference genome. BMC Bioinform..

[38] Wang T., Li B., Nelson C.E., Nabavi S. (2019). Comparative analysis of differential gene expression analysis tools for single-cell RNA sequencing data. BMC Bioinform..

[39] Leinonen R., Sugawara H., Shumway M., International Nucleotide Sequence Database Collaboration (2011). The sequence read archive. Nucleic Acids Res..

[40] Dewick P.M. (2002). Medicinal Natural Products: A Biosynthetic Approach.

[41] Thimmappa R., Geisler K., Louveau T., O’Maille P., Osbourn A. (2014). Triterpene biosynthesis in plants. Annu. Rev. Plant Biol..

[42] Cochrane G.R., Galperin M.Y. (2010). The 2010 nucleic acids research database issue and online database collection: A community of data resources. Nucleic Acids Res..

[43] Finn R.D., Attwood T.K., Babbitt P.C., Bateman A., Bork P., Bridge A.J., Chang H.-Y., Dosztányi Z., El-Gebali S., Fraser M. (2017). InterPro in 2017—Beyond protein family and domain annotations. Nucleic Acids Res..

[44] Janocha S., Schmitz D., Bernhardt R. (2015). Terpene hydroxylation with microbial cytochrome P450 monooxygenases. Biotechnology of Isoprenoids.

[45] Song Y., Yang X., Yang S., Wang J. (2019). Transcriptome sequencing and functional analysis of Sedum lineare Thunb. Upon salt stress. Mol. Genet. Genom..

[46] Prall W., Hendy O., Thornton L.E. (2016). Utility of a phylogenetic perspective in structural analysis of CYP72A enzymes from flowering plants. PLoS ONE.

[47] Cao J., Wang B., Tan X. (2019). Transcriptomic responses of the clam Meretrix meretrix to the organophosphorus pesticide (dimethoate). Ecotoxicology.

[48] Kuhad R.C., Deswal D., Sharma S., Bhattacharya A., Jain K.K., Kaur A., Pletschke B.I., Singh A., Karp M. (2016). Revisiting cellulase production and redefining current strategies based on major challenges. Renew. Sustain. Energy Rev..

[49] Jain K.K., Kumar A., Shankar A., Pandey D., Chaudhary B., Sharma K.K. (2019). De novo transcriptome assembly and protein profiling of copper-induced lignocellulolytic fungus Ganoderma lucidum MDU-7 reveals genes involved in lignocellulose degradation and terpenoid biosynthetic pathways. Genomics.

[50] Bugg T.D., Ahmad M., Hardiman E.M., Rahmanpour R. (2011). Pathways for degradation of lignin in bacteria and fungi. Nat. Prod. Rep..

[51] Li F., Ma F., Zhao H., Zhang S., Wang L., Zhang X., Yu H. (2019). A white-rot fungal lytic polysaccharide monooxygenase drives the degradation of lignin by a versatile peroxidase. Appl. Environ. Microbiol..

[52] Dimarogona M., Topakas E., Olsson L., Christakopoulos P. (2012). Lignin boosts the cellulase performance of a GH-61 enzyme from Sporotrichum thermophile. Bioresour. Technol..

[53] Esquerré-Tugayé M.-T., Boudart G., Dumas B. (2000). Cell wall degrading enzymes, inhibitory proteins, and oligosaccharides participate in the molecular dialogue between plants and pathogens. Plant Physiol. Biochem..

[54] Miyara I., Shafran H., Kramer Haimovich H., Rollins J., Sherman A., Prusky D. (2008). Multi-factor regulation of pectate lyase secretion by Colletotrichum gloeosporioides pathogenic on avocado fruits. Mol. Plant Pathol..

[55] Drori N., Kramer-Haimovich H., Rollins J., Dinoor A., Okon Y., Pines O., Prusky D. (2003). External pH and nitrogen source affect secretion of pectate lyase by Colletotrichum gloeosporioides. Appl. Environ. Microbiol..

[56] Tardy F., Nasser W., Robert-Baudouy J., Hugouvieux-Cotte-Pattat N. (1997). Comparative analysis of the five major Erwinia chrysanthemi pectate lyases: Enzyme characteristics and potential inhibitors. J. Bacteriol..

[57] Tang Y., Wu P., Jiang S., Selvaraj J.N., Yang S., Zhang G. (2019). A new cold-active and alkaline pectate lyase from Antarctic bacterium with high catalytic efficiency. Appl. Microbiol. Biotechnol..

